# Observations on Laue diffraction within synchrotron radiation and neutron macromolecular crystallography research and developments

**DOI:** 10.1063/4.0000225

**Published:** 2023-12-15

**Authors:** John R. Helliwell

**Affiliations:** School of Chemistry, University of Manchester, Manchester M13 9PL, United Kingdom

## Abstract

A seminal contribution in the domain of physiologically relevant biological structure and function determination was by Keith Moffat, of Cornell and latterly of the University of Chicago proposing that synchrotrons should offer the option of a Laue method data collection mode. I enthusiastically joined in supporting this initiative. This proposal needed detailed methods development though; theoretical, experimental and software development. This work was added to the broad research and development program of synchrotron radiation at the UK's SRS. This whole program led to knowledge transfer from the UK's SRS to the ESRF as well as for neutron Laue protein crystallography to the reactor spallation sources and later to spallation neutron sources.

## INTRODUCTION

In 1974 when I was interviewed for my DPhil position at the Laboratory of Molecular Biophysics in Oxford University by the Head, Professor David Phillips, he showed me a flow cell (Wyckoff *et al.* 1967) to initiate a reaction in an enzyme via diffusion of substrate into the crystal. It was an inspiring moment for me as a young and aspiring physics graduate to enter the field in the Oxford Laboratory. During my DPhil I learnt that the x-ray protein crystallography data collection times were incredibly long however compared to an enzyme's functioning time. Later as synchrotron beam line scientist at the UK's SRS 9.6 (Helliwell et al 1986) I could provide greatly reduced x-ray protein crystallography data collection times to support the pioneering studies of the glycogen phosphorylase enzyme “catalysis in the crystal” study (Hajdu *et al.,* 1987a). A seminal contribution in the domain of physiologically relevant biological structure and function determination was by Keith Moffat, of Cornell and latterly of the University of Chicago proposing that synchrotrons should offer the option of a Laue method data collection mode (Moffat *et al.,*1984). I enthusiastically joined in supporting this initiative as it completely broadened the Wyckoff *et al.* (1967) vision by encompassing much shorter exposure times. This synchrotron Laue diffraction proposal (Moffat *et al.,*1984) needed detailed methods development though spanning theoretical, experimental and software development. This R&D work was added to the broad research and development program of synchrotron radiation protein crystallography that I described in 1979 at the Daresbury Study Weekend organized by Ian Munro (Manchester University) and Bob Cundall (Salford University). I gave the opening lecture there and set forward my ideas and an action plan for protein crystallography instrumentation, methods, and applications at the upcoming first ever dedicated x-ray synchrotron radiation source the SRS (Helliwell, 1979). I had several themes: high resolution diffraction data; large unit cells; dynamical studies [i.e., via a Wyckoff *et al.* (1967) flow cell to initiate a reaction in an enzyme]; and phase determination using the two-wavelengths anomalous dispersion phasing method of Hoppe and Jakubowski (1975). Each theme though involved monochromatic diffraction data collection. Later, I learnt from Professor Charlie Bugg, who I had met during his sabbatical in Oxford in 1975 with my DPhil supervisor Dr. Margaret Adams, that at Cornell was Professor Keith Moffat. In 1984 I learnt about his initiative on Laue diffraction and protein crystals aiming at shortening the required exposure times that may be required for time-resolved protein crystallography (Moffat *et al.*, 1984). We were commissioning SRS wiggler 9.6 protein crystallography beamline at the time; a summary is provided in Helliwell *et al.* (1986). Within this I had pressed the mechanical engineer, Phil Moore, as well as the Daresbury Laboratory radiation safety officer, to have a straight through operating position on SRS 9.6 to insert a double crystal monochromator for a faster wavelength change than was achievable with the single bounce triangular monochromator. On SRS 9.6 the predominant monochromator type was the latter due to the large SRS source size of 14 × 0.4 mm^2^ but meant a slow tuning of the x-ray wavelength. Unfortunately, the SRS had not been conceived and designed for providing a small focus beam onto a protein crystal of typically 0.3 × 0.3 mm^2^. In fact, in 1985 the SRS was closed to install a high brightness lattice (the HBL) which did improve the SRS source size to about 2 × 0.3 mm^2^. This was however a long way from what soon became the availability of an ESRF design where even an ESRF bending magnet improved on the SRS HBL source sizes, and associated beam intensity, by an important factor of 10, on top of which were the benefits of x-ray undulator intensities at the sample immediately up by about 1000 on what we had at SRS 9.6 fed by the 3 pole superconducting wiggler. A key question put to me at the early ESRF workshop in 1983 or so was “Would your protein crystals withstand the thermal and radiation blast of the ESRF x-ray beam?”. How to answer this question? The answer of course was to use the SRS wiggler white beam in protein crystal Laue diffraction as a mimic of the expected monochromatic x-ray intensities from the ESRF undulator (Hedman *et al.,* 1985).

So, Moffat *et al.* (1984)'s initiative of Laue diffraction meant that I could immediately make tests on protein crystals at SRS 9.6 in the straight through position which did yield white beam Laue diffraction Helliwell (1984). Secondly, ESRF was obviously some years away from build and beamlines' commissioning. So, to join in with the much higher intensities of the white beam in Laue diffraction could be very interesting for the dynamical studies theme at SRS. Furthermore, by then, the research team of Professor Louise Johnson as PI and Dr. Janos Hajdu as lead researcher were conducting marvelous catalysis in the crystal studies on SRS 9.6 of the enzyme phosphorylase b Hajdu *et al.* (1987a). So, for me to learn about Laue diffraction potentially had a wide impact on the user experimental program.

Professor Keith Moffat and I got in contact, and he came for a year to the UK funded by the Guggenheim Foundation Fellowship, ardently supported by Professor David Blow at Imperial College, London. We focused on theoretical considerations of the fundamentals of Laue diffraction for typical protein crystal unit cells. We promptly learnt that Laue diffraction spots had a majority proportion of single not multiple reflections, as asserted by Bragg (1975). So, even in broad bandpass mode it was appropriate for us to have discussions of wavelength normalization of the measured intensities to put all the measured data onto a common intensities scale.

### Collaborations with Keith and Durward Cruickshank: The multiplicity and angular distribution of reflections in Laue diffraction

In this collaboration we first investigated the multiplicity distribution in Laue diffraction. Crucially we developed an understanding of how this varied with wavelength bandpass. This was a crucial step as the dominant assumption in Moffat *et al.* (1984) was that a wavelength bandpass of around 0.2 was essential to record singlet only reflection containing Laue diffraction spots. In Sec. 10.2 of Cruickshank *et al.* (1987) our main findings were described: -

*“Perhaps the most striking conclusion is that, even for the widest wavelength range that is readily accessible (say 0.3<λ<3.0Å) the proportion of reciprocal-lattice points that lie on single rays exceeds 83%.”* [From now on in this article the term “reciprocal-lattice points that lie on single rays” are called singlets.]

That: -

*“The remaining 17% are non-randomly distributed in reciprocal space*.”

The first of these two points showed that a relatively straightforward wavelength normalization was possible. In the first instance a scaling of measured Laue intensities of singlets against a monochromatic dataset was practical. More subtly, the recording of symmetry related reflections, or a repeated measurement of the same reciprocal lattice point, if singlets, would allow a wavelength dependent scale factor to be derived Campbell *et al.* (1986).

The second of these two points, that the remaining 17% were non-randomly distributed, would in fact be dominated by low hkl integers. This meant there would be a high probability of an hkl having a 2h2k2l, etc., harmonic of hkl and led to the term “low resolution hole” in the measured singlets' data. To reduce the wavelength bandpass was not a solution to this challenge as the small integer hkls would then not be recorded at all. Therefore, there would remain a challenge of needing a “harmonics deconvolution” analytic method. Diffraction data completeness across the full resolution range, i.e., including low resolution, would be important to preserve a good quality electron density map with continuous features and free of possible noise ripple artifacts. Also, though, as remarked in Moffat *et al.* (1984) “there are other experimental circumstances in which a more restricted wavelength range is advantageous.” This was that the x-ray background under a Laue diffraction spot would increase with a larger wavelength bandpass. This opened a wide-ranging exploration of Laue reflection intensity signal to noise, for securing acceptable precision of the weaker high-resolution reflections, on which electron density map details would depend. Moffat and collaborators (Srajer *et al.*, 2000) settled on narrow bandpasses and conveniently provided by the then new undulator sources at third generation sources. The undulator as well can be tapered to yield somewhat broader wavelength bandpasses albeit not as broad as wigglers or bending magnets. That said exploration of reducing x-ray backgrounds with a steadily improving collimator design have been reported Meents *et al.* (2017) and wider bandpasses can then be considered again.

**FIG. 1.**
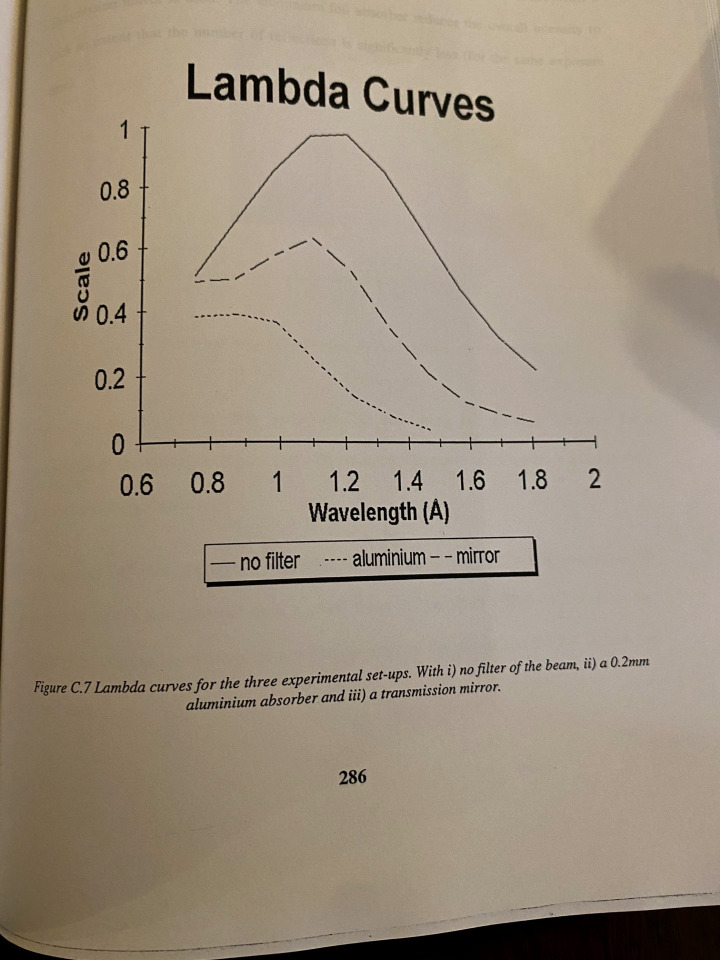
λ curves for the three experimental setups used on SRS 9.5 and a crystal of the protein concanavalin A. (i) no filter of the beam (ii) a 0.2 mm aluminum absorber and (iii) a transmission mirror. From Deacon (1996) page 286 with permission of Dr. Ashley Deacon. The Laue diffraction patterns for each of these three measuring conditions are shown in Fig. 5 of Cassetta *et al.* (1993).

A variable wavelength bandpass, and with adjustable λ_min_ and λ_max,_ was going to be useful. At both SRS protein crystallography high field wiggler beamlines, 9.6 (Helliwell *et al.*, 1986), and later 9.5 (Brammer *et al.*, 1988), focusing platinum coated reflecting mirrors were part of the x-ray optics usually setting λ_min_ at 0.5 Å but could be adjusted. How to adjust λ_max_? The simplest was a thin aluminum foil placed in the beam path to absorb a fraction of the intensity, according to its thickness. A more elegant solution, but involving a design and build, was to have a reflecting mirror of a longer λ_min_ cut off but that was very thin thus letting through the longer wavelengths complementary to the λ_min_ of the reflected x-ray beam of the first mirror. This concept was introduced by Lairson and Bilderback (1982) using a soap film. This was implemented and tested as part of Ashley Deacon's Ph.D. in the University of Manchester Deacon (1996) but using mylar, later followed by a new material “Luxan.” Figure 1 shows the λ curves determined from the Laue diffraction patterns recorded on SRS 9.5 and processed using the Daresbury Laue software (Helliwell *et al.,* 1989) by Deacon (1996) as part of his University of Manchester Ph.D.

Cruickshank *et al.* (1991) explored the theoretical foundations of the spatial distribution of the reflections in Laue diffraction as well. In turn this led to Helliwell (1991) developing a 3D “toastrack” of photographic films. The concept being that the film closest to the protein crystal would subtend a large solid angle and record the longer wavelength reflections, as well as the short wavelength ones. These latter would penetrate the front and subsequent films and gradually expand to fill the last film. This was tested with two versions of the toast rack, constructed in the University of Manchester Chemistry Department workshops, accommodating the two different sizes of CEA photographic film available at the time. Tests at the CHESS white beamline in Cornell University (Helliwell, 1991) demonstrated that this concept also worked with two Kodak storage phosphor sheets placed one behind the other, some distance apart. The 3D toast rack arrangement was reminiscent of the neutron time-of-flight spallation neutron source crystallography which the author became closely familiar with when Chairman of the European Spallation Source Neutron Macromolecular Crystallography Scientific and Technical Advisory Panel (ESS NMX STAP) between 2013 and 2023.

In parallel with these instrumentation and methods developments a collaboration with the Daresbury Computational Division staff, Mike Elder, Pella Machin, and John Campbell, commenced to develop software for the synchrotron Laue diffraction data processing described in Helliwell *et al.* (1989). An important spin-off of the protein crystal Laue diffraction into chemical crystallography was led by Marjorie Harding at Liverpool University (reviewed by Harding, 1995). A whole raft of validation tests was undertaken on the software. These are summarized in Nieh *et al.* (1999). The first electron density map from protein crystal Laue diffraction data were determined by Hajdu *et al.* (1987b).

Michael Wulff of ESRF wanted to develop a Laue diffraction beamline there (ID09) and JRH hosted a visit by Michael at SRS 9.5 (Brammer *et al.*, 1988). This ESRF ID09 planned beamline had far superior intensity to that available at the SRS 9.5. Keith Moffat and collaborators (Bourgeois *et al.*, 1996) undertook pioneering sub nano second Laue diffraction at that ESRF Laue ID09 beamline on time-resolved CO debinding and rebinding in myoglobin, a process stimulated by a laser flash onto a crystal of carefully chosen size to allow penetration of the laser beam through the whole crystal (Srajer *et al.*, 1996). Likewise, JRH undertook a diffusion of substrate into a crystal flow cell experiment on the enzyme hydroxymethylbilane synthase (Helliwell *et al.,* 1998). Overall, the vision of David Phillips explained to me at my DPhil interview in the spring of 1974, showing me a Wyckoff *et al.* (1967) flow cell, I felt had been fulfilled. Meanwhile neutron Laue protein crystallography, anticipated in the study by Cruickshank *et al.* (1987) started to takeoff (Helliwell and Wilkinson, 1994).

### Spin off into neutron Laue diffraction at the Institut Laue Langevin, Grenoble

The theoretical fondations of Laue diffraction laid out by Cruickshank *et al.* (1987) created a major interest at the Institut Laue-Langevin neutron research reactor in its potential for neutron crystallography of proteins and small molecules (Helliwell and Wilkinson, 1994). By a happy coincidence in my laboratory in Manchester I noticed that crystallization of the protein concanavalin A by my research student Susanne Weisgerber using dialysis had led to exceptionally large crystals up to 3 mm long. We undertook our first neutron Laue crystallography data collection at the ILL's new Laue diffractometer (LADI), Habash *et al.* (1997). Thus began a suite of experiments over the next 15 years understanding the hydrogenation and hydration of this protein and its likely relevance to its saccharide binding, explored also by ultrahigh resolution synchrotron x-ray diffraction (Deacon *et al.*, 1997), a back-to-back paper with Habash *et al.* (1997), and a molecular dynamics investigation of the structure and thermodynamics of ligand binding (Bradbrook *et al.*, 1998). These studies were extended by Habash *et al.* (2000) and (Ahmed et al., 2007) as the ILL LADI instrument improved to LADI-III, with an improved sensitivity of its neutron sensitive imaging plate scanner system by reading the inside of the plate not the outside (Blakeley *et al.,* 2010). This innovation was then further advanced by relocation of the LADI-III to a new neutron guide, increasing the neutron fluxes at the protein crystal. Overall reductions in the exposure times per image of approximately 5 were achieved. This gain could be used to extend the diffraction resolution for a fixed exposure time or reduce the crystal sample volume required (see the graphical plot introduced by Dr. Clive Wilkinson at the ILL in Grenoble showing examples of where the new Laue diffractometer (LADI I) instrument can bring into range the more challenging projects [i.e. see Fig. 1 of Habash *et al.* (1997)] and updated for LADI III in Fig. 20 of Blakeley, (2009). These technical factors and the steady improvement of capabilities of neutron protein crystallography have been summarized by Blakeley and Podjarny (2018).

**FIG. 2.**
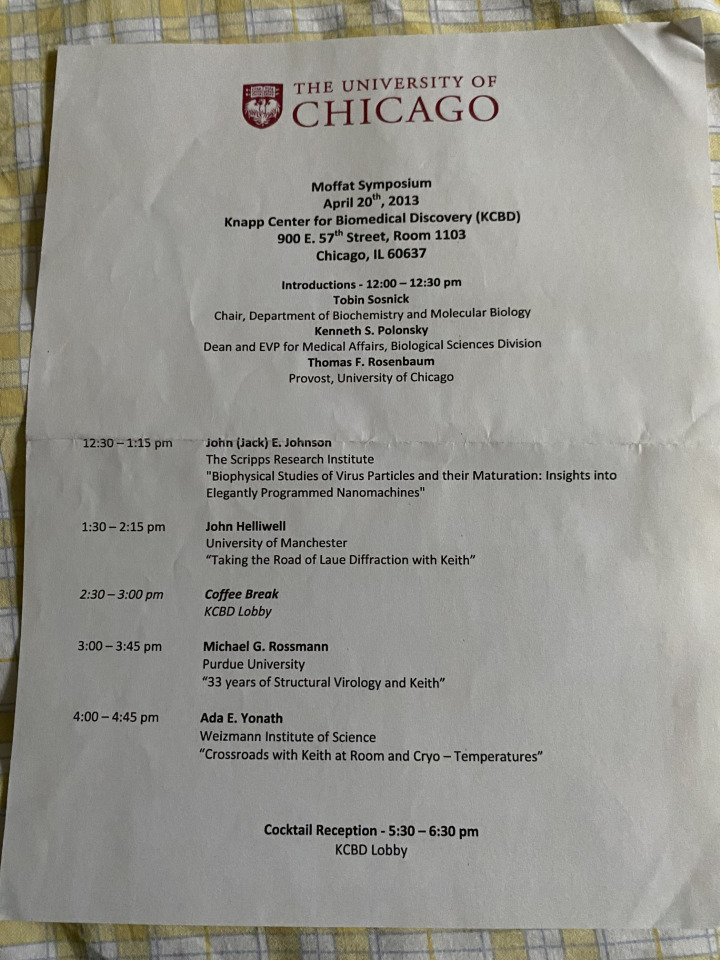
The program of speakers at the Moffat Symposium held in Chicago in April 2013.

It was an honor for me to lecture at the Moffat Symposium in April 2013 describing the developments above; the Symposium program sheet is shown in Fig. 2.

### Spin off into neutron time-of-flight white beam diffraction at the ESS, Lund

**FIG. 3.**
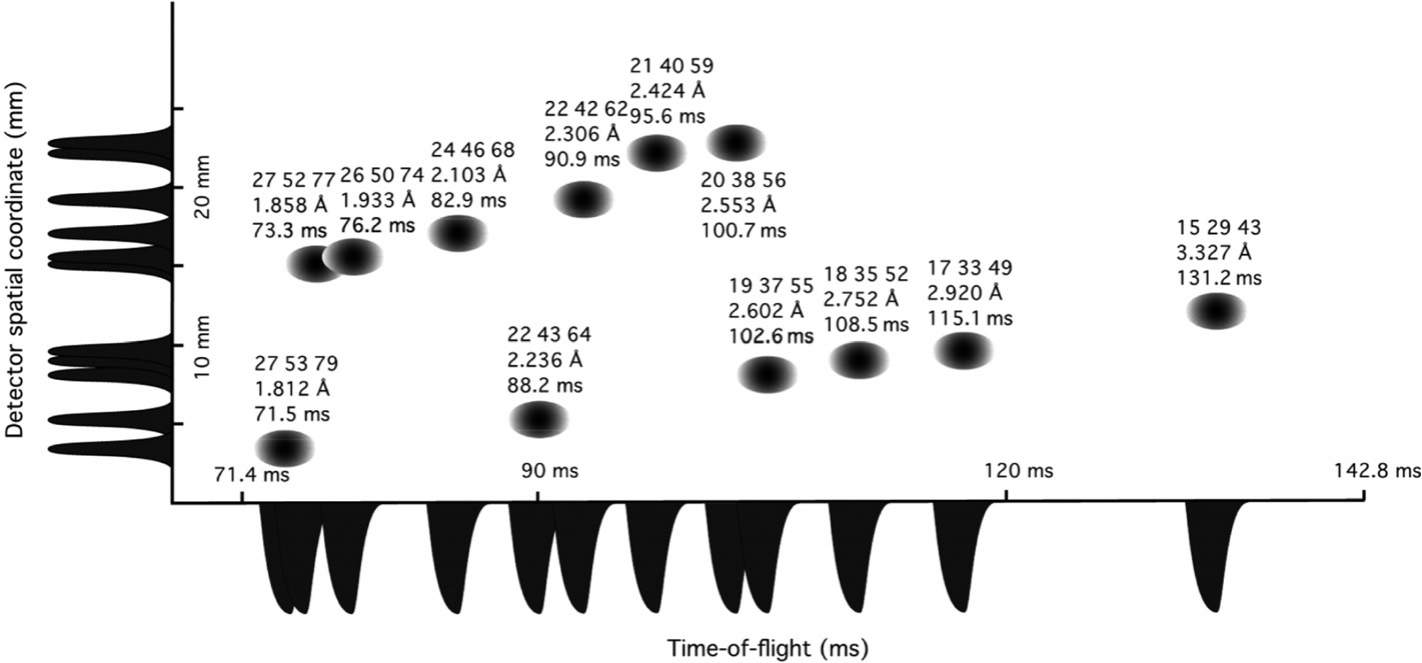
The separation of some simulated example high-resolution reflections from bovine heart cytochrome c oxidase (molecular weight 442 kDa; crystal parameters: space group P2_1_2_1_2_1_, unit cell *a* = 181.8, *b* = 204.1, and *c* = 177.8 Å) at 1 m detector distance in the spatial and time-of-flight dimensions on ESS NMX (from Andersen *et al.* 2020). Most but not all reflections are individually resolved. Reprinted with permission from Andersen *et al.*, Nucl. Instrum. Methods Phys. Res., Sect. A **957**, 163402 (2020). Copyright 2020 Elsevier and Dr. Esko Oksanen of ESS.

The ESS is under construction in Lund. Within this there is a neutron macromolecular crystallography instrument “NMX” led by Esko Oksanen (https://europeanspallationsource.se/instruments/nmx). The instrument design is described here https://europeanspallationsource.se/sites/default/files/downloads/2018/03/NMX_proposal_2013_rev.pdf. A comprehensive description of the ESS facility and its instruments, including NMX, is given in Andersen *et al.* (2020). The time-of-flight spallation source Laue diffraction style of measurement is a 3D distribution involving as well as position sensitivity of the detector a third dimension which is the time of flight of the neutrons in any Laue diffraction spot. This allows then a full bandpass of wavelengths of the neutrons to be harnessed (1.8–10 Å; see Andersen *et al.* 2020; Table 6) with the signal to noise preserved and spatial overlap of the diffraction spots (Cruickshank *et al.*, 1991) relaxed by use of this 3D measurement approach. The instrument design was facilitated by simulating complete diffraction patterns from larger unit cells (NMX_proposal_2013_rev.pdf) combining the McStas Monte Carlo package (Willendrup and Lefmann, 2020) supplemented with simulated Laue patterns using the program Lauegen (Campbell *et al.*, 1998) distributed as part of the Daresbury Laboratory Laue Software Suite (Helliwell *et al.*, 1989) now available for download described at Hao *et al.* (2021). As Andersen *et al.* (2020) describe, the NMX proposed concept has the time width of a reflection of ∼4 ms, a relatively small fraction for each measurement 3D frame (Fig. 3). At the time of writing this article the actual shape of the ESS pulse is yet to be measured which may affect these simulations. The first beam on target i.e., first neutrons at ESS is planned to be 2025. The NMX detector, being made in collaboration with CERN, is described by Pfeiffer *et al.* (2023).

### The broad research and development program for synchrotron radiation protein crystallography at the UK's Daresbury Laboratory

**FIG. 4.**
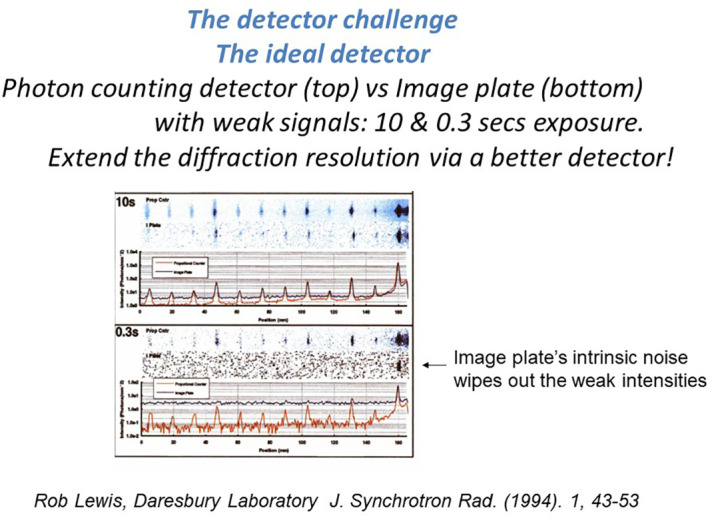
The evolution of detector hardware for measuring x-ray diffraction data. The pixel detector became the culmination of this evolution and dominated by the excellent company Dectris in Switzerland with their range of excellent hardware. This work of my colleague of the time at the Synchrotron Radiation Source at Daresbury Laboratory, UK. Dr. Rob Lewis, showed up what we were striving for in terms of the ideal photon counting detector compared with the best integrating (analogue) detector of the time the image plate (Lewis, 1994).

All the Laue research and development work described above was added to the broad research and development program of synchrotron radiation protein crystallography at Daresbury Laboratory described at its outset in Helliwell (1979;1984). Then, this was all set in the context of the evolution of synchrotron radiation sources, and the impact of crystallography needs on the accelerator and beamline specifications, as well as detectors (Fig. 4), and the growth of its importance as a result within crystallography (Helliwell, 2012 ).

### Protein crystal growth

In parallel with these facility developments over the past 50 years, a new development was in the macromolecular crystal growth technologies. The international macromolecular crystal growth community commenced a highly successful series of biannual conferences, the international macromolecular crystal growth symposia “ICCBM” (see, e.g., Einspahr *et al.*, 2015). Numerous innovations greatly increased the chances of successful crystallization of any chosen macromolecule. The innovations of molecular biology in turn allowed a much more successful production of pure proteins from any gene of interest, academically or for drug discovery. The field of structural genomics was initiated (see, e.g., https://en.wikipedia.org/wiki/Structural_genomics).

The growth of interest in neutron protein crystallography also of time resolved crystallography both emphasized that the functional state of the protein in the crystal should be at or as close as possible to the operational chemical conditions of the living cell (see, e.g., Helliwell, 2020). Therefore, the manipulation of the chemical conditions to realize a successful crystallization could well be problematic for structure and function studies. The chance to manipulate the macromolecular crystallization conditions through the physics of fluids in microgravity offered a new alternative to chemical conditions manipulation (Snell and Helliwell, 2021). This latter, in particular, has been harnessed for neutron protein crystallography as the protein crystal volumes realized can be substantially enhanced (see, e.g., Drago *et al.*, 2022).

### Concluding remarks and outlook

For x-rays and time-resolved crystallography, Laue diffraction it seems has in the past decade been rather overtaken by the x-ray laser serial monochromatic crystallography method, known as serial femtosecond crystallography “SFX.” A resurgence beckons however in the adoption of serial crystallography at synchrotron radiation sources (‘SSX') for time-resolved crystallography. These sources are undergoing a major refurbishment of the machine to be “extremely bright sources” (see ESRF EBS at https://www.esrf.fr/home/UsersAndScience/Accelerators/ebs-extremely-brilliant-source.html). By adding in the use of polychromatic beams there are further reductions of exposure times (Meents *et al.*, 2017). There is then a closing of the shortest possible exposure times, i.e., a capability gap, in time-resolved capabilities between the x-ray laser SFX end stations and the EBS synchrotron source SSX new beamlines. Since there are many more synchrotrons with SSX capabilities (actual or planned) than x-ray lasers with SFX end stations the adoption of x-ray Laue diffraction can have a renewal. There always remains the challenge of suitable protein crystal systems for time-resolved crystallography and of means of reaction initiation. These challenges have challenged the whole field for decades. However, the interest in time-resolved crystallography has grown and is growing further as crystallographers see that protein fold predictions are made with a confidence often adequate to commence the protein crystal structure determination procedure by molecular replacement as an alternative to use of multiple x-ray wavelengths first demonstrated with two different metal anode laboratory x-ray tubes by Hoppe and Jakubowski (1975) and who also discussed their seminal study in the context of synchrotron radiation, first implemented at the Stanford SSRL led by Keith Hodgson (Phillips *et al.,* 1976, Phillips *et al.,* 1977, 1980). See also the important formalism of Okaya and Pepinsky (1956). The development of a “general phasing vehicle” of selenomethionine proteins was a major addition to the use of multiple x-ray wavelengths initiated by Hendrickson *et al.* (1990) and Hendrickson (1999). The intercontinental expansion of beamlines provision for multiple x-ray wavelengths for phasing and element identification using resonant scattering was a major development described by Cassetta *et al.* (1999). All these developments led to a sustained increase in the number and type of entries in the Protein Data Bank summarized by Abad-Zapatero (2012).

The adoption of Laue diffraction by neutron sources for macromolecular crystallography described above is buoyant both at reactor and spallation sources worldwide. It has also been harnessed for chemical crystallography at the Institut Laue Langevin with its VIVALDI instrument initiative (Cole and McIntyre, 2004; Wilkinson *et al.*, 2009) and at ANSTO with its Koala instrument (https://www.ansto.gov.au/our-facilities/australian-centre-for-neutron-scattering/neutron-scattering-instruments/koala-laue). The use of monochromatic neutron beams for protein crystallography at FRM II is a notable exception and is a successful active operation (Ostermann and Schrader, 2015).

Overall, the freedom to harness polychromatic beams that Moffat *et al.* (1984) introduced has been a very significant initiative. To join in with the important initiative of Keith Moffat of synchrotron Laue diffraction has been an exciting and worthwhile part of my scientific research career. Thank you, Keith.

## A PARALLEL THREAD IN LAUE DIFFRACTION CHEMICAL CRYSTALLOGRAPHY

In the concanavalin A collaboration described in this article I visited the Weizmann Institute twice, hosted by Aaron Joseph Gilboa and Joseph Yariv. I lectured in the “Gerhard Schmidt Lecture Theatre” (https://www.weizmann.ac.il/ConstructionEngineering/catalog/collaboration-spaces/gerhard-schmidt-lecture-hall). I asked who Gerhard Schmidt was? I was told that he was a pioneer of combining crystallography with chemistry and photochemistry such as the photochemical changes in nitrophenol (Coppens and Schmidt, 1965). See biographical sketch https://en.wikipedia.org/wiki/Gerhard_Schmidt_(crystallographer). Furthermore, in the same visit I met Dov Rabinovich who explained to me his interest in developing Laue diffraction for such studies pioneered by Gerhard Schmidt. His paper Rabinovich and Lourie (1987) and references cited therein is of considerable interest to the parallel development of synchrotron Laue diffraction. Another parallel development in Laue chemical crystallography, at the SRS, for use in time dependent studies, of small specimens, in twinning diagnostics and in pressure cells was described by (Wood et al., 1983).

## Data Availability

The data that support the findings of this study are available from his depositions in the Protein Data Bank: 1YPN, 2YPN, 1AH5, 1NLS, 1GIC, 242D, 1C57, 1XQN, 2YZ4, 1OBQ and 1OBU.
